# Prices of blood units in South East Asia

**DOI:** 10.4103/0973-6247.42690

**Published:** 2008-07

**Authors:** N. Choudhury

**Affiliations:** *Medical Director, Prathama Blood Center, Vasna, Ahmedabad - 380 007, India*

The question of appropriate price tag for a unit of blood has been a subject of controversy over a long time. People in general are of the opinion that there cannot be any price for a free gift donated by a blood donor. They are absolutely right and there should not be any price for blood and components. But why do various countries charge money for a unit of blood? Actually, this is not the price for blood but service charges for processing blood units after collection. It includes collection cost like blood donor motivation, donor selection, cost of bag, donor refreshment; testing cost like confirmation of ABO/Rh, mandatory transfusion associated infection tests; component preparation cost; preservation cost like maintaining refrigerators, deep freezers, platelet shakers/ incubators; infrastructure cost like maintenance of equipments/ gadgets, electricity, salary of employees, other consumables and office expenses.

This is not as simple as it sounds. The blood banking community has often been tarnished by words like ‘profit’, ‘commercial’ and ‘sale’ etc. These are supposed to be ‘dirty’ words in blood banking. Blood units are donated voluntarily. These activities have made general public averse to pricing on blood units. ‘Service charge’ is often interpreted as ‘selling price’ with ‘profitable’ motive. Nobody should engage in trading of liquid human tissue i.e. blood. The basic question is that should there be any service charge for blood and components? The answer is ‘yes’. Though blood is received through a selfless act of donation, there is cost involved in the whole operation as mentioned earlier. This cost has to be borne by somebody. Whether it is the government, health insurance company or the patient, depends upon the health services availed by the patient. Honorable Supreme Court of India passed the judgment (1996) that “…These (blood units) will be screened, processed and distributed as blood components to local hospital based blood centers against service charges…”. It has again been echoed in the Indian National Blood Policy (2002) stating that, “The mechanism shall be introduced in government sector to route the amounts received through cost recovery of blood/ blood components to the blood banks for improving their services” (objective:2, para:2.2.3).

If we see historical part of pricing of blood and components in India, National AIDS Control Organization (NACO) passed a circular in 1992 that for government hospital’s service charge should be Indian rupees (INR) 250 and for private hospitals it should be INR.500 (present exchange rate of about Indian Rs.38= 1 US$). During last fifteen years, the price has not been revised officially. During this period, anti HCV test has been included which alone costs about INR.50-90 per test. Of course, some expenditure like single blood bag cost has come down from an average of INR.60 to INR.40 due to cutthroat competition. However, we should not forget about other increased costs like sales taxes, overheads expenses like salary, electricity, diesel etc. Then what should be the price for a unit of blood? This burning problem was referred to the Technical Resource Group (TRG) of the NACO to submit suggestions in 2004. After deliberation, it was recommended that the service charge of one unit of whole blood or RBC should not be more than INR.655. This charge is the maximum charge suggested after conforming minimum standard prescribed by the Drug and Cosmetic act (1999). If anybody does extra tests for the benefit of the patient, (for example anti HBc, ALT etc.) probably that cost may be added as per unit cost to INR.655. This recommendation was never implemented but in 2006, National Blood Transfusion Council (17^th^ NBTC meeting) suddenly came out with one pricing structure. They suggested that price of blood components should be fixed as: whole blood at INR550, packed red cells at INR400, plasma at INR200, platelet (random) at INR200 and cryo precipitate at INR100 We do not know the basis of such pricing which seems to be highly unrealistic. However, realizing the incorrect nature of this decision, NBTC immediately withheld this pricing from implementation. NBTC subsequently did a wise thing by constituting a committee to re-look into pricing.

Latest recommendations by NBTC forwarded on 7^th^ November 2007 were as follows: whole blood and red cells: INR.850, fresh frozen plasma: INR.400, platelet concentrate (random): INR.400 and cryoprecipitate: INR.200. There is no absolute truth in deciding these figures. For example, NBTC suggested that salary for all staff in blood bank should be managed under INR. 120 per unit multiplying total units collected per year. What is the basis of these figures? How can anybody decide salary of professionals/ individuals working in blood banks in private/ NGO or even government sector? After recent hefty salary increase in government sector (INR.10,000 for lowest entry level) is this type of costing going to work? How does one fix the price for blood bank consumables? It is directly related to market economy. For example, when this exercise was taken up, crude oil price was probably US$ 85/ barrel which is at present hovering around US$ 140. You know that plastic blood bags are petroleum by products. However, you may also argue that price may also decrease as we have seen that price of anti HCV tests reduced from Rs.100 per test to INR.30 per test in last decade. Another issue is that if the blood bank wants to employ additional tests or measure for blood safety, the price will increase. For example, if one blood bank wants to start Nucleic Acid test (NAT), price will increase by INR.900 to INR.1600 per unit depending upon tests performed on single or pooled samples. Likewise, if any blood bank starts laboratory leukoreduction, price of blood unit will rise by another couple of hundred rupees.

It is the truth that there is a wide variation in service charges in different parts of India. There is no uniformity in charges among blood banks run by the government, Indian Red Cross, NGO and private hospitals. Charges differ in various government hospitals of various states or even in same states. The same is applicable for other categories of blood banks. A small study was carried out four years back on various service charges of about 35 premier Transfusion Medicine centers of India operating as stand-alone or hospital-based blood banks. The highest charges of these blood banks have been calculated because minimum charges of almost all blood banks are free units without any charges. It has been observed that mean service charge of RBC unit is INR.705.32 (range: INR350 to INR.1350). Majority of blood banks have different charge structure for various economical classes. The mean cost of FFP is INR. 539.28 (range: INR.250 to INR.900). Though, cryoprecipitate is used for hemophilia patients, it costs a maximum of INR.250 to INR.900 (mean: 526.73). Many hospitals have discounted charges for hemophilia patients on wet cryoprecipitate units. The mean service charge of random donor platelet is INR.497.32 (range: INR.400 to INR.900), however single donor platelets cost a maximum of INR.14,500 per unit. What are the present service charges? Most of the blood banks have increased their service charges. To best of my knowledge, one unit of NAT tested and leukoreduced RBC costs more than INR.4000.

Let us look around other countries in this part of the world. There are few countries which have organized blood transfusion services (BTS) and others have fragmented BTS like India. One of the countries that has made good progress in quality standard is Sri Lanka. BTS in this country is organized and about 95% blood supply is through government controlled system. There is no charge for any blood or components supplied through this system to government system. If government BTS supplies to private health care system, then they charge Sri Lankan rupees 560 (SL Rs.140= 1 US$) for red cell units. The same charge is levied for FFP and random donor platelets. However, aphaeresis platelet costs about SL Rs.14,000. About 5% blood supply is distributed through private health care providers and the cost of blood and components are significantly high. One unit of RBC is about SL Rs.3,000-4,000 which is about six times higher than government run BTS. Another organized BTS exits in Bhutan. There is only government run BTS and all blood and components are issued without any charges to all hospitals. All expenses are borne by the Royal Bhutan government.

If we look towards other nearby countries, Pakistan has fragmented BTS like India. There are two provinces, Sind and Punjab where the transfusion services are better organized. The situation in the rural areas is not well developed. The situation in Baluchistan and North West Frontier Province need improvement. BTS can be divided into few categories i.e. government, Red Crescent Societies, NGO, private and small profit making blood banks. The quality standard of government blood bank is gradually improving. There are no charge for blood and components by government blood banks if used inside government hospitals. NGO blood banks are operating on non for profit motive and they usually charge between Rs.600 to 800 (about 72 Pakistani rupees= 1 US$) plus ask for a family replacement donor for any component or whole blood. The private hospitals mainly operate by collecting blood from family relatives and there is no uniform criterion for charges on blood components. The charges range between Rs 1200 to 1800 hundred per unit of RBC. Sometimes, charges may go up to Rs.3000 depending upon emergency situations and blood bank supplying components. There are multiple small blood banks which are actually run by technicians and have just fulfilled the requirements of the blood transfusion authority. The charges are between Rs.500 to Rs.1000 and this amount may go up in different situations and in rare blood groups.

Bangladesh also has a fragmented BTS and there are blood banks in government, NGO, Red Crescent and corporate sectors. In the government hospitals, the charge for blood for general beds is Taka 250, for paying beds it is Taka 350 and Taka 500 (about 70 Taka= 1 US$) when a patient is admitted in a special room. In private sectors, Taka 500-600 are charged per whole blood units and components are charged as Taka 600-800 per units. Nepal is another country from this region. It has fairly organized BTS and it is primarily managed by Nepalese Red Ross Society. Service charges of blood and components are different in government and private sectors. When whole blood is supplied to public sector hospitals, Nepalese Rs.410 is charged but Rs.610 is charged from private sectors hospitals (about 80 Nepalese Rs.= 1 US$). When fresh packed red cells are supplied, Rs.685 is charged from public hospitals and Rs.910 is charged from private hospitals.

Different types of BTS exist in South East Asian countries. All are developing economies. However, cost recovery for blood and components are different. It is a welcome move if the government can take care of all cost involved in BTS but it may not be possible for all countries to supply blood free of cost. There are definite costs involved in this operation. If general population can afford to pay or it is paid by the insurance companies, there should a cost recovery system to sustain and maintain the BTS. Special attention should be paid so that safe blood supply to under privileged population is continued. Government and Transfusion Medicine specialists from this part of the world should pay special attention so that there should be a reasonable cost recovery system and no body should make unwanted profit from the gift of blood. Forceful price control may not work in developing economy. Stringent price control by the government may force BTS to compromise with quality system and supply of safe blood. Some guidelines may be helpful for fragmented BTS of these countries. But guidelines should be a reviewed frequently (may be once in a year) so that BTS should withstand economic pressure of raising inflation.

